# Characterization of Chronic Cutaneous Lesions from TNF-Receptor-1-Deficient Mice Infected by *Leishmania major*

**DOI:** 10.1155/2012/865708

**Published:** 2011-12-08

**Authors:** Carolina Ferreira Oliveira, Daniel Manzoni-de-Almeida, Paula Seixas Mello, Caio Cotta Natale, Helton da Costa Santiago, Luíza da Silva Miranda, Fernanda Oliveira Ferraz, Liliane Martins dos Santos, Mauro Martins Teixeira, Rosa Maria Esteves Arantes, Leda Quercia Vieira

**Affiliations:** ^1^Departamento de Bioquímica e Imunologia, Instituto de Ciências Biológicas, Universidade Federal de Minas Gerais, Avenue Antonio Carlos 6627, 31270-901 Belo Horizonte, MG, Brazil; ^2^Núcleo de Pesquisa em Ciências Biológicas, Instituto de Ciências Biológicas e Exatas, Universidade Federal de Ouro Preto, Morro do Cruzeiro, 35400-000 Ouro Preto, MG, Brazil; ^3^Departamento de Patologia Geral, Instituto de Ciências Biológicas, Universidade Federal de Minas Gerais, Avenue Antonio Carlos 6627, 31270-901 Belo Horizonte, MG, Brazil

## Abstract

*Leishmania major*-infected TNF receptor 1 deficient (TNFR1 KO) mice resolve parasitism but fail to resolve lesions, while wild-type mice completely heal. We investigated the cell composition, cytokine production, and apoptosis in lesions from *L. major*-infected TNFR1 KO and wild-type (WT) mice. Chronic lesions from *L. major*-infected TNFR1 KO mice presented larger number of CD8+ T and Ly6G+ cells. In addition, higher concentrations of mRNA for IFN-**γ** CCL2 and CCL5, as well as protein, but lower numbers of apoptotic cells, were found in lesions from TNFR1 KO mice than in WT, at late time points of infection. Our studies showed that persistent lesions in *L. major*-infected TNFR1 KO mice may be mediated by continuous migration of cells to the site of inflammation due to the presence of chemokines and also by lower levels of apoptosis. We suggest that this model has some striking similarities to the mucocutaneous clinical form of leishmaniasis.

## 1. Introduction

Parasites of the genus *Leishmania *cause a spectrum of cutaneous manifestations ranging from limited cutaneous lesions that heal spontaneously to the more severe mucocutaneous form. These different clinical manifestations depend on the species of *Leishmania* and the host immune response [1, 2]. In experimental models, it is established that BALB/c mice are susceptible to infection by *Leishmania major*. This mouse strain develops progressive lesions, uncontrolled growth of parasites, visceralization, and death. The C57BL/6 strain is resistant to infection by *L. major*, controls parasite replication, and heals lesions [3]. However, despite clinical and pathological cure of the disease, the parasite remains latent in the host. Resistance to infection by *L. major* is mediated by IFN-*γ*, TNF-*α*, and activation of macrophages to produce nitric oxide [4–6].

Mucocutanesous leishmaniasis is caused mainly by *L. braziliensis*. It is characterized by control of parasite growth in the tissue, but persistent chronic inflammation that commonly affects mucosal tissue causing severe disfiguration and social stigma to the patient [7]. High concentrations of inflammatory cytokines, namely, IFN-*γ* and TNF-*α*, are found in these patients [8]. The study of mucocutaneous leishmaniasis is hampered by lack of a good experimental model. Experimental infection with *L. braziliensis* causes a very mild and self-limited lesion in C57BL/6 and BALB/c strain mice [9, 10]. In addition, *L. amazonensis* causes a persistent chronic lesion in C57BL/10 and C57BL/6 mice that lasts over 20 weeks, but both animal models fail to control the parasite in the tissue, a hallmark of mucocutaneous leishmaniasis. Moreover, IFN-*γ* and TNF production is impaired in these infection models [11–13]. The closest animal model for mucocutaneous disease would be the infection of C57BL/6 TNFR1-deficient (TNFR1 KO) mice with *L. major.* TNFR1 KO mice control tissue parasitism similarly to the wild-type resistant mouse, but develop nonhealing lesions. However, these lesions do not increase in size progressively. On the contrary, they remain chronic and small, but last for at least 20 weeks afterinfection [14, 15]. 

In experimental infection by *L. major,* TNF-*α* is important for activation of macrophages, in cooperation with IFN-*γ*, and elimination of intracellular parasites [4, 5, 16–18]. Another important fact is the involvement of TNF in the induction of apoptosis in lymphocytes from lesions from wild-type *L. major*-infected mice [19]. This information may suggest that TNF-*α* may play a key role in the healing of *L. major *lesions. However, this important phenomenon was not described in the chronic stage of infection to explain the persistent lesions in *L. major*-infected TNFR1 KO. Thus, the aim of this study was to characterize events in the chronic phase of *L. major* infection in TNFR1 KO mice. 

## 2. Materials and Methods

### 2.1. Animals

 C57BL/6 wild-type (WT) mice, 6 to 10 weeks old, were obtained from CEBIO (Universidade Federal de Minas Gerais, Belo Horizonte, MG, Brazil). TNFR1 KO mice were originally obtained from the University of Pennsylvania (Philadelphia, Pa, USA, a kind gift from Dr. Phillp Scott and Dr. Klaus Pfeffer) and maintained in Laboratory of the Gnotobiology and Immunology of the Instituto de Ciências Biológicas (UFMG, Brazil). All the procedures involving animals were in accordance with the ethical principles in animal research adopted by the Brazilian College of Animal Experimentation and were approved by the UFMG animal experimentation ethical committee at UFMG (CETEA), protocol number 55/2009.

### 2.2. Parasites and Infection

A clone of *Leishmania major* (WHO MHOM/IL/80/Friedlin) was used in this study. Parasites were maintained in Grace's insect medium (GIBCO BRL Life Technologies, Grand Island, NY, USA), pH 6.2, supplemented with 20% fetal bovine serum (Nutricell, Campinas, SP, Brazil), 2 mM l-glutamine (SIGMA Chemical Co., St. Louis, Mo, USA), 100 U/mL penicillin and 100 *μ*g/mL streptomycin (GIBCO BRL Life Technologies). Mice were injected in the hind footpads with 1 × 10^6^ *L. major* metacyclic promastigotes. Footpads were measured weekly with a caliper (Mitutoyou, Suzano, SP, Brazil). Lesion sizes are expressed as the difference between infected and uninfected footpads.

### 2.3. Parasite Load

Parasite load in infected footpads was determined by limiting dilution [14]. Results were expressed as the negative log of the last positive dilution.

### 2.4. Histological Analyzes

Infected footpads from WT and TNFR1 KO mice were removed at 6 and 15 weeks after infection and fixed in 10% of formalin. Tissues were processed and embedded in paraffin and 5 *μ*m thick sections were stained with hematoxylin and eosin and analyzed by light microscopy. At least 10 microscopic fields measuring 250,000 micrometers, representative of lesions, were automatically analysed by KS 300 (Carl Zeiss, Germany) for determining the comparative cellularity of lesions at 15 wks.

### 2.5. Apoptosis Analysis

Infected footpads from WT and TNFR1 KO mice were removed at 6 and 11 weeks after infection and fixed in 4% formaldehyde. Apoptotic cells were assessed *in situ* by the TUNEL reaction, an *in situ* cell death detection kit (POD, Roche Applied Science, Penzberg, Germany). The results were obtained by counting the number of stained cells per 100 cells (600 cells counted per animal) in 6–10 random areas per histological section.

### 2.6. Flow Cytometry

Infected footpads from WT and TNFR1 KO mice were removed at 6 and 15 weeks after infection. Collected tissues were incubated for 90 minutes with 1.5 mg/mL of collagenase (Sigma-Aldrich, Mo, USA) in RPMI 1640 without supplements at 37°C, homogenized using a tissue grinder and centrifuged at 2000 g. Single-cell suspensions were stained with fluorochrome-conjugated antibodies (eBioscience, San Diego, Calif, USA) against CD4 (RM4-5), CD8 *α* (53-6.7), CD3 (17A2), F4/80 (BM8), and Ly6G (RB6-8C5) in PBS containing 1% FBS for 20 min on ice and then washed and fixed with 2% formaldehyde. Stained cells were analyzed using an FACScan flow cytometer equipped with cellQuest software (Becton Dickinson, Heidelberg, Germany). Statistical analyses of mean fluorescence intensity (MFI) were performed using the FlowJo v7.6.5 software (Tree Star Inc., Ashland, Ore, USA).

### 2.7. Chemokines and Cytokines Analysis

Chemokines and cytokines were analyzed by two methods: detection of mRNA by reverse transcription polymerase chain reaction (RT-PCR) and ELISA, at different times of infection (1 and 2 days, 2, 6, and 11 weeks). The footpad was excised and total protein and RNA were extracted with Trizol (GIBCO BRL Laboratories), as previously described. Cytokine and chemokine detections by RT-PCR were performed as previously described [20]. Briefly, *L. major*-infected and noninfected footpads were harvested and placed in 0.5 mL TRIZOL (GIBCO BRL) solution and homogenized, and RNA extraction was performed according to the manufacture's instruction. RNA was quantitated spectrophotometricaly and 1 *μ*g of RNA was reverse transcribed using 25 U of M-MLV Reverse Transcriptase (Promega Corp., Madison, Wis, USA) in 12.5 *μ*L reaction containing 2.5 mM dNTPs, 50 mM TRIS/HCl (pH8.3), 75 mM KCl, 2 mM MgCl_2_, 10 mM DTT (GIBCO BRL Laboratories, Grand Island, NY, USA), 250 mM dNTP (Promega), 50 mM Tris-HCl, pH 8.3, 75 mM KCl, 3 mM MgCl_2_, 10 mM DTT, 10 U RNAsin (GIBCO BRL), and 7.5 pM of oligo dT_15_ (GIBCO BRL). The mixtures were incubated for 5 min at 95°C, 5 min at 4°C, and 5 min at 25°C; at this step 25 U of reverse transcriptase was added to each sample and the reaction mixture was incubated for 60 min at 37°C. The temperature was then elevated to 95°C for 5 min and cooled again at 4°C for 5 min. The PCR was performed in 10 *μ*L reaction of samples diluted in the following buffer: 250 mM dNTP, 10 mM Tris-HCl pH 8.3, 50 mM KCL, 1.5 mM MgCl_2_, 5 mM of each primer and 0.05 U of TaqPolimerase (Phoneutria, Belo Horizonte, MG, Brazil). For each pair of primers, at least three different numbers of cycles were tested and the best nonsaturating condition was chosen. The PCR was standardized using the house-keeping gene HPRT. The primer sequences (sense and antisense sequence), PCR product size, number of cycles, and temperature of annealing used for each primer are listed in Table 1. The products were electrophoresed in 6% polyacrilamide gels and developed by silver staining. Primers and PCR conditions are listed in Table 1. For ELISA, footpads were homogenized in PBS (0.4 M NaCl and 10 mM de Na_2_HPO_4_, pH 7.2) containing antiproteases (0.1 mM phenylmehtylsulfonyl fluoride, 0.1 mM benzethoinium chloride, 10 mM EDTA, and 20 mM KI aprotin A/100 mL) and 0.05% tween-20. Homogenized tissues were kept on ice for 30 min and subsequently centrifuged (3000 × g for 10 min). The preparations were assayed using RD Systems kits (Duoset-RD Systems, Minneapolis, Minn, USA) according to instructions. The sensitivity for CCL-2 and CCL-5 was 16 and 32 pg/mL, respectively.

## 3. Results

**Figure 1:**
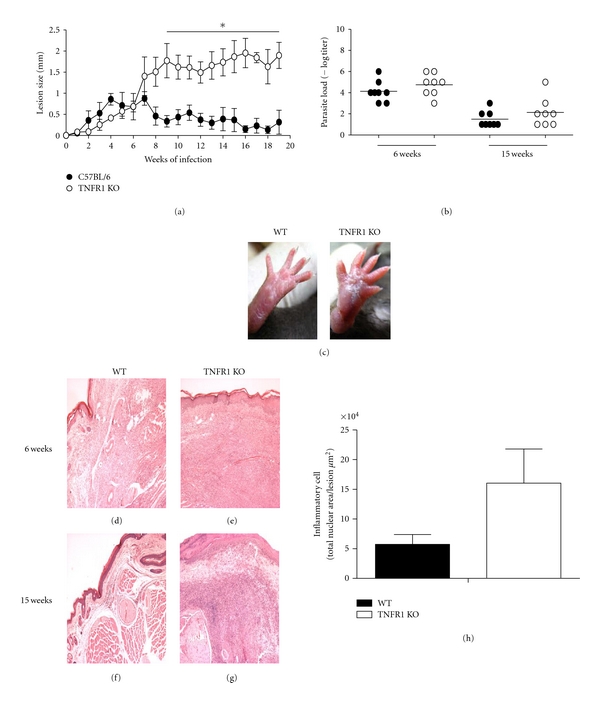
Lesions and parasite burdens in TNFR1 KO mice with *L. major*. Mice were infected with 1 × 10^6^ metacyclic stationary forms of *L. major* in footpads. (a) The footpads were measured weekly and the value for uninfected mice was subtracted from each infected footpad to estimate lesion size. (b) Parasite burden in WT and TNFR1 KO mice. Mice were sacrificed at 6 and 15 weeks afterinfection and parasite burden was determined by limiting dilution analysis (*n* = 5 mice per time point). (c) Lesions from WT and TNFR1 KO mice infected with *L. major *15 weeks afterinfection. (d–g) Histological aspect of tissues from lesions from WT and TNFR1 KO infected for 6 weeks (d and e) or 15 weeks (f and g), stained with hematoxylin and eosin. (h) Morfometric quantification of inflammatory cells from WT and TNFR1 KO lesions 15 weeks afterinfection (*n* = 4 mice per group). *P < 0.05. Data are from one experiment of three performed independently.

In this work, we characterized the lesions in *L. major-*infected TNFR1 KO mice and attempted to explain the reason why these lesions fail to heal. As shown previously [14] *L. major*-infected TNFR1 KO mice develop chronic nonhealing lesions, while WT mice heal lesions over time (Figure 1(a)). In addition, as previously described [14], parasite growth is controlled at the site of infection (Figure 1(b)). WT mice had completely healed lesions at 15 weeks of infection; however, TNFR1 KO mice had conspicuous lesions that led to some loss of function (Figure 1(c)). When histological examination of lesions was performed, we found that, albeit at 6 weeks of infection the inflammatory infiltrate was similar in both groups, at 15 weeks very few inflammatory cells were observed in WT mice, while lesions from TNFR1 KO displayed a high number of inflammatory cells (Figures 1(d)–1(h)). 

**Figure 2:**
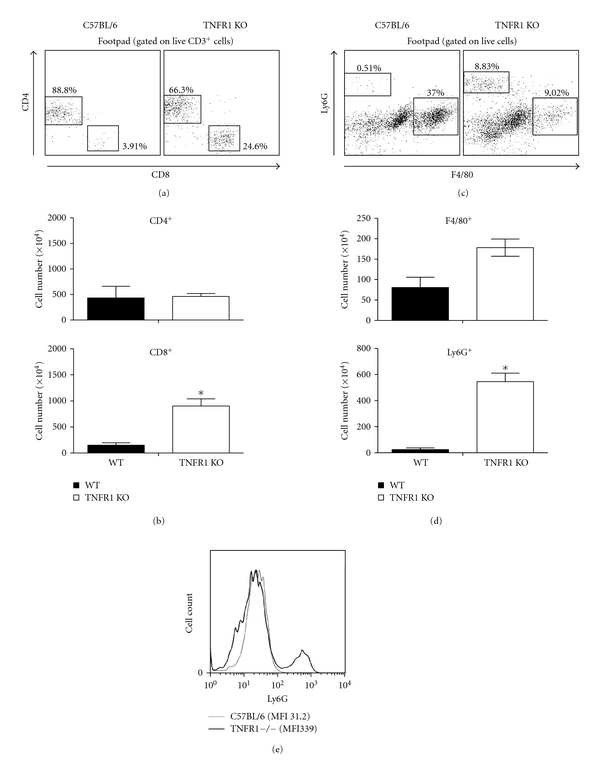
Cell populations in lesions from WT and TNFR1 KO mice infected with *L. major*. Mice were infected in the footpad with 1 × 10^6^ *L. major* and 15 weeks post-infection the inflammatory cells were isolated from lesions and characterized by flow cytometry. (a) Dot plots and (b) absolute cell numbers of CD4+ and CD8+ T cells from pooled lesions from 4 mice per group. (c) Dot plots and (d) absolute cell numbers of F4/80+ and Ly6G+ cells from pooled lesions from 4 mice per group. (e) Ly6G fluorescence intensity. *P < 0.05. Data are from one experiment.

We further characterized the inflammatory infiltrate by flow cytometry. As seen in Figure 2(a), a higher percentages of CD4+ T lymphocytes was found in lesions from WT mice at 15 weeks of infection. However, since there were more cells in the inflammatory infiltrate in TNFR1 mice, when we calculated the absolute numbers of cells, similar numbers of CD4+ cells were observed in lesions from both groups of mice (Figure 2(b)). Higher percentages and absolute numbers of CD8+ T cells were found in lesions from TNFR1 KO mice (Figures 2(a) and 2(b)). Also, at 15 weeks of infection a higher percentage of macrophages (F4/80 positive cells) was found in WT mice (Figure 2(c)), while a higher percentage of Ly6G positive cells was found in TNFR1 KO mice (Figure 2(c)). In absolute numbers, we found similar numbers of CD4+ T cells and F4/80 cells in both groups of mice (Figure 2(d)) while a higher absolute number of Ly6G+ cells were found in lesions from TNFR1 KO mice (Figure 2(d)). In addition, expression of Ly6G on a per cell basis, indicated by the mean fluorescence intensity (MFI), was increased in these mice (Figure 2(e)).

**Figure 3:**
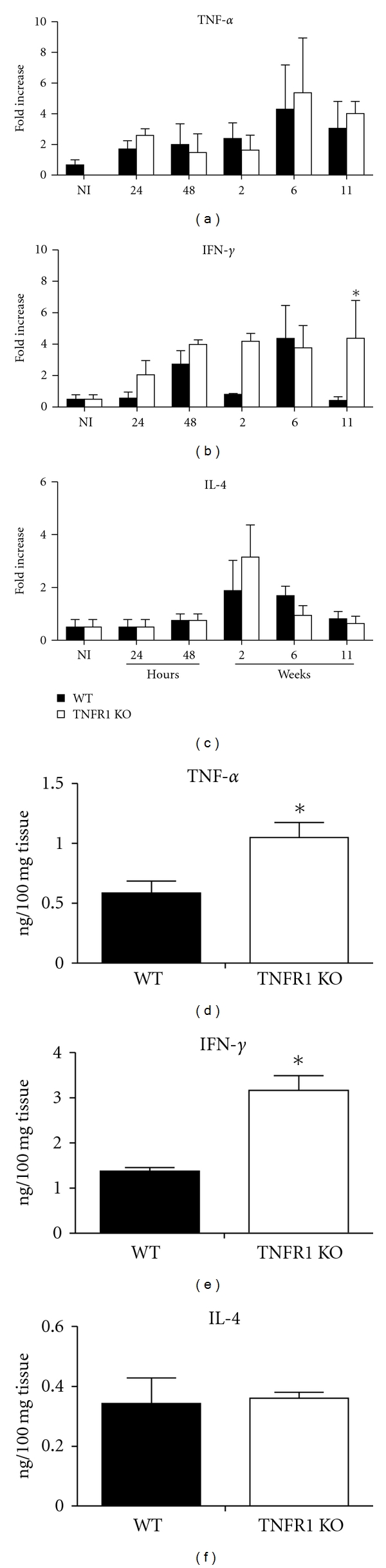
TNF-*α*, IFN-*γ*, and IL-4 in lesions from WT and TNFR1 KO mice infected with *L. major*. Mice were infected in both footpads with *L. major* (1 × 10^6^). At 24 and 48 h and at 2, 6, and 11 weeks after infection, mice were killed, lesions were harvested, and RT-PCR reactions performed for cytokines. At 15 weeks protein was assayed by ELISA. (a–c) Fold increase in TNF-*α*, IFN-*γ*, and IL-4 mRNA, obtained by densitometry of bands in gels, as described in Section 2. (d–f) TNF-*α*, IFN-*γ*, and IL-4 protein was determined by ELISA 15 weeks after infection. Data shown are from one experiment representative of two indenpendently performed ones, with four mice per group. *P < 0.05.

We proceeded to investigate the expression of cytokines at the site of infection. As seen in Figure 3(a), similar concentrations of mRNA for TNF-*α* were found in both groups of mice. Similar concentrations of IFN-*γ* mRNA were found in lesions from both groups at 24 and 48 hours of infection, but higher concentrations were found in lesions from TNFR1 KO mice at 11 weeks of infection (Figure 3(b)). In addition, similar concentrations of IL-4 message were found in both groups at all time points tested. These data were confirmed by ELISA at 15 weeks of infection. We found higher TNF-*α* and IFN-*γ* protein concentrations in *L. major*-infected TNFR1KO mice (Figures 3(d) and 3(e)) than in WT mice. No differences were found for IL-4 protein expression between groups of mice (Figures 3(c) and 3(f)). 

**Figure 4:**
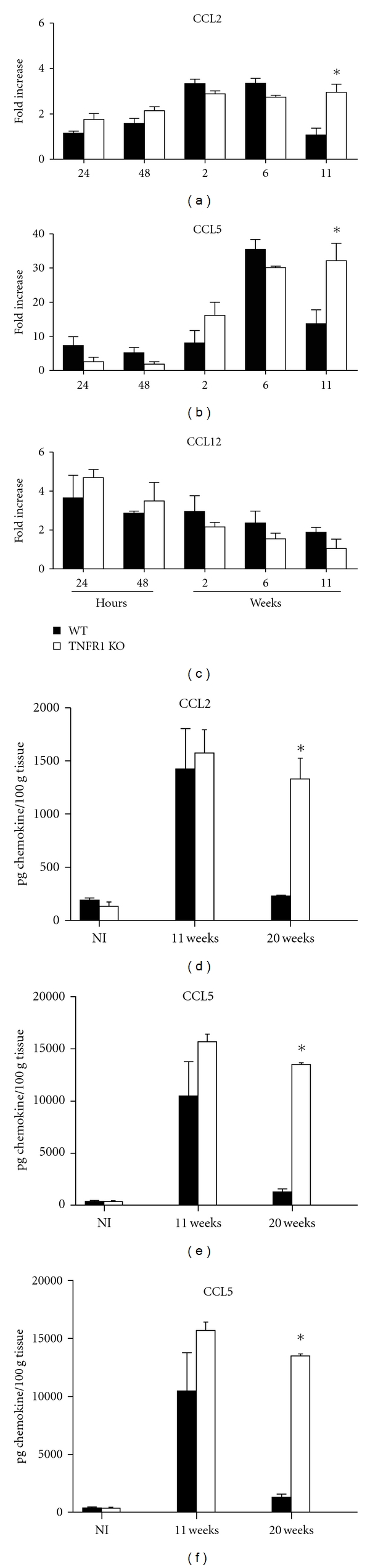
CCL2, CCL12, and CCL5 in lesions from WT and TNFR1 KO mice infected with *L. major*. Mice were infected in both footpads with *L. major* (1 × 10^6^). At 24 and 48 h and at 2, 6, and 11 weeks after infection, mice were killed, lesions were harvested and RT-PCR reactions were performed for chemokines. At 15 weeks, protein was assayed by ELISA. (a–c) Fold increase for CCL2, CCL12, and CCL5 mRNA, obtained by densitometry of bands in gels, as described in Section 2. (d–e) CCL2 and CCL5 determined by ELISA in footpads of naïve mice (NI), and mice infected for 11 and 20 weeks. Data shown are from a single experiment representative of two separate experiments performed with four mice per group. *P < 0.05.

Since there were more cells at the site of infection in TNFR1 KO mice, we investigated the expression of chemokines by RT-PCR and ELISA. When we assayed CC cytokines, as seen in Figure 4, we found that at 11 weeks of infection there was higher expression of CCL2 and CCL5 mRNA in *L. major*-infected TNFR1 KO mice than in WT mice (Figures 4(a) and 4(b)). No differences were found in expression of CCL12 mRNA between the groups of mice (Figure 4(c)). We confirmed the results found for CCL2 and CCL5 by ELISA and found higher concentrations of protein (Figures 4(d) and 4(e)) at 20 weeks of infection in lesions from TNFR1 KO mice. In addition, no differences at later time points were found for CXCL1, CXCL9, and CXCL10 mRNA (data not shown). 

**Figure 5:**
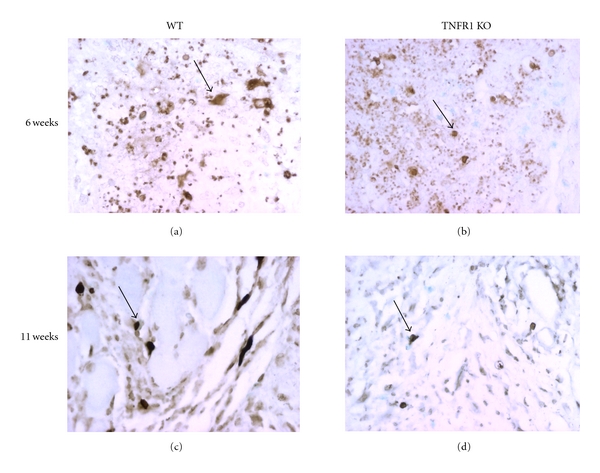
Apoptosis in lesions from WT and TNFR1 KO mice infected by *L. major*. Mice were infected with *L. major *in the footpad (1 × 10^6^) and after 6 weeks (a and b) and 11 weeks (c and d) of infection. Tissues were stained by TUNEL, as described in Section 2. Arrows indicate TUNEL positive cells.

In addition to more recruitment, a larger cellular population at the site of infection may be explained by a defective removal of inflammatory cells. Since TNFR1 mediates apoptosis [19, 21], we investigated apoptosis at the site of infection at 6 and 11 weeks by the TUNEL reaction. As seen in Figure 5 and quantified in Table 2, at six weeks of infection we saw TUNEL-positive cells in both TNFR1 KO and WT mice. However, at 11 weeks of infection we found higher numbers of TUNEL positive cells in WT mice.

## 4. Discussion

Cutaneous leishmaniasis is characterized by lesions that are self-limited and may heal spontaneously over time. However, approximately 3% of infected individuals develop the mucocutaneous form of the disease, with chronic ulcerative lesions affecting mucosal tissues and cartilage. One of the features in mucocutaneous leishmaniasis in humans is the prolonged maintenace of lesions [7, 22]. These tissues present an inflammatory infiltrate rich in mononuclear and polymorphonucelar cells [23–26]. High concentrations of IFN-*γ*, TNF-*α*, and IL-17 are found in affected tissues and are produced by peripheral blood monocuclear cells from these patients [8, 26, 27]. Another feature is a deficiency in the regulation of the inflammatory processes in mucocutaneous patients, when compared to patients who developed the cutaneous form of the disease [27]. Tissue damage is caused apparently by the inflammatory response, since parasites are detected in low concentrations or not at all in lesions [23–25, 28]. In this work, we studied the chronic cutaneous lesion developed by *L. major*-infected TNFR1 KO mice. This model presents some of the features found in mucocutaneous patients: control of parasite replication, high concentrations of IFN-*γ* and TNF-*α*, and a large uncontrolled inflammatory infiltrate. Hence, *L. major*-infected TNFR1 KO mice can control parasite replication, but do not overcome the intense inflammatory process that is observed even after 20 weeks of infection. Thus, lesions in *L. major*-infected TNFR1 KO mice present some similarities to mucocutaneos lesions in patients infected (Table 2). This model may be useful for studies aimed at interfering with the development of chronic nonhealing lesions caused by *Leishmania.*

Resolution of *L. major *growth in mice is mediated by nitric oxide produced by nitric oxide synthase 2 (NOS2), which is induced by IFN-*γ* and TNF-*α* [4–6]. Given the high concentrations of IFN-*γ* and TNF-*α* present in lesions from TNFR1 KO mice infected with *L. major, *it is not surprising that these mice control parasite growth. The reason for discrepancy between mRNA and protein expression of TNF-*α* is not known, but we could speculate that there is posttrancriptional regulation or that protein is accumulated at the site of infection, since there is no signaling through TNFR1. Albeit TNF-*α* was shown to signal through TNFR1 to induce NOS*2*, alternative pathways in TNFR1 KO mice have been proposed [15, 29, 30]. In fact, mice deficient in both receptors for TNF are capable of controlling parasite burdens [15], and it has been proposed that direct contact with T cells (via CD40L and LFA1) would substitute for TNF-*α* [29]. Thus, as previously described [14, 15], we show here that persistence of lesions in TNFR1 KO is not mediated by the persistence of parasites at the site of infection. 

TNF-*α* is the starting factor that mediates cell infiltration to a site of infection or tissue damage [21]. Accordingly, it has been shown that in an HSV-1 encephalites mouse model, TNFR1 mice present a smaller cellular infiltrate in response to tissue damage [31]. In other models, it has been shown that TNFR1 KO mice do not control the inflammatory infiltrate and damage tissue [19, 30, 32–35]. In this work, we characterized the cellular infiltrate in lesions from TNFR1 KO mice. We found that, at later times of infection, there were more neutrophils and CD8+ T cells in lesions from TNFR1 KO than in WT mice. CD8+ T cells have been implicated in the exacerbation of tissue injury in patients suffering from mucocutaneous form of leishmaniasis [36–38]. Likewise, the presence of neutrophils has been associated with a high concentration of IL-17 in areas of tissue damage in patients infected with *L. braziliensis* who developed mucocutaneous leishmaniasis [25, 26]. We also found lower percentages of F4/80-expressing macrophages in lesions from TNFR1 KO mice at 15 weeks afterinfection. This seems to be due to the presence of other cells in the same gate (e.g., neutrophils), since absolute numbers of macrophages were similar in both groups of mice. However, the smaller percentage of macrophages (which is not a smaller absolute number) did not compromise the control of parasites by TNFR1 KO mice. 

Cell recruitment to sites of infection or tissue damage is mediated by chemokines [39]. In an attempt to explain the larger cellular infiltrate in TNFR1 KO mice, we determined the concentrations of mRNA for chemokines at several times of infection. At the earlier times of infection the concentrations of the chemokines assessed were similar in both groups. This observation is in accordance with our previous observations that initial cellular recruitment is not affected by the TNFR1, in a model of angiogenesis [40]. However, at 11 weeks of infection, when lesions were significantly different, we found higher expression of CCL5 and CCL2 mRNA and, at 20 weeks afterinfection, we found higher concentrations of CCL5 and CCL2 protein in lesions. This could be simply due to a larger number of cells at the site of infection; however, this is unlikely, since for CCL12 and other chemokines (not shown) we failed to observe the same effect. CCL5 and CCL2 are implicated on the migration of macrophages, monocytes, NK, and T cells to sites of injury [41–43]. Thus, it may be that the persistence of exacerbated inflammatory cells at the site of infection with *L. major* in TNFR1 KO is related with high concentrations of these two chemokines that are promoting the recruitment of more cells. 

Apoptosis is required for the clearance of inflammatory cells from tissues, once the stimulus for recruitment of pathogens or damage is gone [44, 45]. TNFR1 mediates apoptosis, since it contains a cell death domain [46, 47]. In fact, it had previously been shown that there was defective apoptosis at the site of infection of *Rhodococcus equis*. Moreover, cells from *R. equis*-infected lungs of WT mice and from footpads of *L. major*-infected WT were sensitive only to TNF-mediated apoptosis. However, cells from TNFR1 KO mice infected with either *R. equis* or *L. major* were not sensitive to TNF-*α*-mediated apoptosis [19]. Thus, we investigated apoptosis, *in situ*, in lesions from WT and TNFR1 KO mice. At 6 weeks of infection, both mouse strains showed similar concentrations of TUNEL positive cells, thus suggesting that at this time point apoptosis occurred independently of the TNFR1. However, at 11 weeks of infection, when WT mice were healing and TNFR1 KO mice had a large inflammatory infiltrate at the site of infection, lesions from WT mice had significantly more TUNEL positive cells than lesions from TNFR1 KO mice. This indicated to us that TNFR1 plays a crucial role in the clearance of the inflammatory infiltrate and in the healing of lesions. 

## 5. Conclusion

In this paper, we implicated persistent chemokine production and defective apoptosis as the factors that prevent healing of lesions in TNFR1 KO mice. In addition, characterization of parasite burden, cellular infiltrate, and cytokine production allowed us to propose the TNFR1 KO infected with *L. major *as a model to study chronic nonhealing lesions found in patients that present the mucocutaneous clinical form of leishmaniasis (Table 3). 

## Figures and Tables

**Table 1:** Primers, temperature, and number of cycles used for RT-PCR.

Primer	Sequence	Length (bp)
HPRT	58°C 32 cycles	217
	FW GTT GGA TAC AGG CCA GAC TTT GTT	546–569
	RV GAT TCA ACT TGC GCT CAT CTT AGG C	763–739

IL-4	60°C 33 cycles	292
	FW TTT GAA CGA GGT CAC AGG AG	195–214
	RV TGC TCT TTA GGC TTT CCA GG	487–468

IFN-*γ*	60°C 32 cycles	202
	FW GGT GAC ATG AAA ATC CTG CAG	290–310
	RV GCG CTG GAC CTG TGG GTT GTT GAC C	493–469

TNF-*α*	62°C 30 cycles	300
	FW CGC TCT TCT GTC TAC TGA AC	293–312
	RV TGT CCC TTG AAG AGA ACC TG	593–574

CCL2	62°C 30 cycles	488
	FW CCG GAA TTC CAC TCA CCT GCT GCT ACT CAT TCA	179–205
	RV CCG GAA TTC GGA TTC ACA GAG AGG GAA AAA TGG	667–644

CCL5	54°C 26 cycles	309
	FW CGC GGA TCC CCA CGT CAA GG A GTA TTT CTA CAC C	184–209
	RV CGC GAA TCC CTG GTT TCT TGG GTT TGC TGT TG	493–473

CCL12	54°C 29 cycles	380
	FW GTT CCT GAC TCC TCT AGC TTT C	11–32
	RV ACG TAA GAG TTT TTG GAA CTC	391–371

CXCL9	54°C 30 cycles	398
	FW GAT CAA ACC TGC CTA GAT CC	302–321
	RV GGC TGT GTA GAA CAC AGA GT	700–681

CXCL10	62°C 27 cycles	383
	FW CGC GGA TCC TGA GCA GAG ATG TCT GAA TC	250–271
	RV CGC GGA TCC TCG CAC CTC CAC ATA GCT TAC AG	633–611

CXCL1	62°C 34 cycles	521
	FW CGC GGA T:CC TGG ACC CTG AAG CTC CCT TGG TTC	226–251
	RV CGC GGA TCC: CGT GCG TGT TGA CCA TAC ATT ATG	731–708

**Table 2:** Quantification of apoptosis at the site of infection by *L. major* in WT and TNFR1 KO mice.

	C57BL/6 ^a^		TNFR1 KO	
	Mean	Standard error	Mean	Standard error
6 weeks	**7.04** ^b^	0.72	**6.75**	1.00
11 weeks	**8.15** ^c^	1.01	**2.91**	0.52

^a^Mice were infected with *L. major *in the footpad (1 × 10^6^) and after 6 and 11 weeks of infection animals were sacrificed and the lesions collected. Tissues were stained by TUNNEL, as described in Section 2. Apoptotic nuclei were counted and results expressed as apoptotic nuclei per 100 nuclei. Results represent the mean of 3–6 animals; six sections were counted per mouse.

^b^ *P* = 0.37 Student's *t-*test.

^c^ *P* = 0.0005 Student's *t-*test.

**Table 3:** Comparison between human mucocutaneous and TNFR1 KO lesions.

Characteristic	Mucocutaneous leishmaniasis	*L. major*-infected TNFR1 KO
Chronic cutaneous lesions	Yes	Yes
Intense inflammatory cells infiltrate in chronic phase	Yes	Yes
Low parasite load in lesions	Yes	Yes
High production of proinflammatory cytokines (IFN-*γ* and TNF-*α*) in the lesions	Yes	Yes
Defect in the regulation of inflammatory response	Yes	Yes
Presence of high numbers of CD8+ T cells and neutrophils in lesions	Yes	Yes
High levels of IL-17	Yes	Not determined
Invasion of mucosal tissues	Yes	No
